# Synthesis and characterization of quaternary La(Sr)S–TaS_2_ misfit-layered nanotubes

**DOI:** 10.3762/bjnano.10.111

**Published:** 2019-05-24

**Authors:** Marco Serra, Erumpukuthickal Ashokkumar Anumol, Dalit Stolovas, Iddo Pinkas, Ernesto Joselevich, Reshef Tenne, Andrey Enyashin, Francis Leonard Deepak

**Affiliations:** 1 Department of Materials and Interfaces, Weizmann Institute, Rehovot 76100, Israel; 2 Nanostructured Materials Group, Department of Advanced Electron Microscopy, Imaging and Spectroscopy, International Iberian Nanotechnology Laboratory (INL), Avenida Mestre Jose Veiga, Braga 4715-330, Portugal; 3 Chemical Research Support Department, Weizmann Institute, Rehovot 76100, Israel; 4 Ural Federal University, Institute of Mathematics and Computer Sciences, Turgeneva Str. 4, 620083 Ekaterinburg, Russian Federation,; 5 Institute of Solid State Chemistry, Ural Branch of Russian Academy of Sciences, Pervomayskaya Str. 91, Ekaterinburg 620990, Russian Federation

**Keywords:** aberration-corrected STEM, DFT, misfit-layered compounds, nanotubes, Raman spectroscopy

## Abstract

Misfit-layered compounds (MLCs) are formed by the combination of different lattices and exhibit intriguing structural and morphological characteristics. MLC Sr*_x_*La_1−_*_x_*S–TaS_2_ nanotubes with varying Sr composition (10, 20, 40, and 60 Sr atom %, corresponding to *x* = 0.1, 0.2, 0.4 and 0.6, respectively) were prepared in the present study and systematically investigated using a combination of high-resolution electron microscopy and spectroscopy. These studies enable detailed insight into the structural aspects of these phases to be gained at the atomic scale. The addition of Sr had a significant impact on the formation of the nanotubes with higher Sr content, leading to a decrease in the yield of the nanotubes. This trend can be attributed to the reduced charge transfer between the rare earth/S unit (La*_x_*Sr_1−_*_x_*S) and the TaS_2_ layer in the MLC which destabilizes the MLC lattice. The influence of varying the Sr content in the nanotubes was systematically studied using Raman spectroscopy. Density functional theory calculations were carried out to support the experimental observations.

## Introduction

Since their discovery in 1992 [1], inorganic nanotubes (INTs) have attracted the interest of many researchers due to their electrical, optical, mechanical and thermoelectric properties [2] derived from their unique structure. WS_2_ nanotubes are nanostructures originating from the bending of a single layer of the 2D material tungsten disulfide along one axis, resulting in the characteristic high-aspect-ratio morphology typical of these species [1]. The formation of WS_2_ nanotubes is attributed to the instability of the dangling bonds at the periphery of nanometric WS_2_ sheets forcing it to fold into a seamless hollow structure under appropriate conditions [2]. Their morphology permits different functionalities to be combined and is characterized by the presence of different regions corresponding to the inner and outer surfaces (e.g., for adsorption and catalysis), the interstitial galleries (for intercalation), and the tube termination, which could be either opened or capped [3]. Typically, these nanostructures are synthesized by means of high-temperature reactions that allow the formation of different metal sulfide nanotubes [4–5]. Another type of hollow nanostructure, inorganic fullerene-like structures (IFs), is the result of bending of a 2D layer of WS_2_ or any other 2D material along two directions, resulting in a closed-cage quasi-spherical nanostructure [6]. Once available in large quantities [7–8], different electrical devices based on single WS_2_ and MoS_2_ nanotubes could be realized, including high-performance field effect transistors (FETs) [9–10] and electromechanical resonators [11–13]. Using ionic liquid gating, ambipolar p–n junctions led to high-performance light-emitting diodes (LEDs) and photovoltaic devices [14]. Most interesting, however, was the demonstration of quasi-1D superconductivity, which reflected the non-centrosymmetric structure of the chiral WS_2_ nanotubes [15–16]. Remarkably, also IF–WS_2_ NPs were found to be an excellent solid-state lubricant with numerous lubricating and metal-working fluids commercially available with rapidly expanding markets.

More recently, nanotubes of misfit-layered compounds (MLCs) formed by the association of layers from two different kinds of lattices were reported. Numerous MLCs from 2D oxide and chalcogenide compounds were reported in the past. Chalcogenide-based MLCs have the chemical formula (MX)_1+_*_y_*(TX_2_)*_m_* (where M = Sn, Pb, Bi, Sb, rare earth elements; T = Ti, V, Cr, Nb, Ta; X = S, Se; 0.08 < *y* < 0.28, *m* = 1–3) [17–32], denoted for simplicity as MX–TX_2_. The two layers, i.e. MX and TX_2_ with distorted rock-salt and hexagonal structures, respectively, alternate periodically along the *c*-axis. Sometimes more complex MLC superstructures are formed, such as MX–TX_2_–TX_2_, etc. [17–20]. The (distorted) rock-salt layer consists of two atomic planes. In the hexagonal TX_2_ lattice, the metal M atom is sandwiched between two chalcogen (X) atoms, in a trigonal bi-prism (2*H*) or octahedral (1*T*) coordination. The MX and TX_2_ layers are stacked together via van der Waals forces. Frequently, the difference in the work function between the MX and TX_2_ slabs leads to a partial charge transfer from the MX slab to that of TX_2_. This charge transfer induces polar interactions between the layers juxtaposing on the van der Waals forces [29]. As the constituting compounds usually exhibit their own symmetry and space groups, their unit cells differ from each other along, at least, one direction. Therefore, MLCs are incommensurate and do not have a unit cell. They are often represented for simplicity as an approximant, made usually of 5 MX and 3 TX_2_ units along the *a*-axis, respectively. Also, due to the different unit-cell volume of the two components, the (MX)_1+_*_y_*TX_2_ are non-stoichiometric compounds with the term 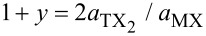. Interestingly, several chromium sulfide based compounds of the form MCrS_3_ where reported early on, however their misfit structure was understood only many years later [33–34]. Importantly also, the hexagonal CrS_2_ (VS_2_) is not a stable polymorph unless it is intercalated in the galleries of the van der Waals gap by an electron donor (Lewis base). Incidentally, in [32] the authors mention that: “Another type of crystals with a "hollow-rod" shape often grow in a same batch”. Unfortunately, the authors did not elaborate any further or study these “hollow-rod” shaped crystallites in their following work.

Nanotubes based on rare-earth monosulfide-tantalum disulfide MLCs have been the subject of a few works, which demonstrate the possibility of synthesizing such structures using both early and late lanthanides on the scale of tens of milligrams. This product could then be analyzed and its properties were studied beyond the nanoscale [35]. It has been recently demonstrated that extension of this synthetic protocol allows the introduction of several types of heteroatoms, yielding bulk MLCs which were not reported in the literature hitherto, and nanotubes thereof. The first example of this new strategy was dedicated to the synthesis of LnS–TaSe_2_ nanotubes, which exhibit double periodicity La/Ta and S/Se superstructures [36]. Here, the nanotubes (NTs) are characterized by the presence of a superstructure in which the Se atoms preferentially occupied the hexagonal crystal positions around the Ta atoms, while the sulfur atoms showed a preference for the rock-salt sites in the LnS lattice [36]. More recently, the inclusion of Nb atoms in LaS–TaS_2_ nanotubes has been studied in detail allowing the identification of various Nb-rich structures in which the heteroatoms replace the Ta occupying the hexagonal sites in the TaS_2_ lattice [37]. An outstanding observation in the Nb-rich LaS–Nb*_x_*Ta_1−_*_x_*S_2_ nanotubes was the appearance of a periodicity with interlayer (*c*-axis) spacing of 2.35 nm instead of the expected 1.18 nm. This double periodicity was attributed to a superstructure with each two sequential LnS layers (and possibly also the Nb*_x_*Ta_1−_*_x_*S_2_ layers) rotated 30° (60° in the ortho-pseudohexagonal unit cell) with respect to each other. Furthermore, instead of the stable 2*H*-TaS_2_ (2*H*-NbS_2_) [35,38] polytype, the Nb-rich LaS–Nb*_x_*Ta_1−_*_x_*S_2_ slabs were all found to be in the 1*T* state, i.e., with octahedral coordination of the Ta(Nb) atoms in the Nb*_x_*Ta_1−_*_x_*S_2_ lattice. The charge transfer from the M (rare earth element) to the Ta atom of TaS_2_ in MS–TaS_2_ MLC has been discussed in the past [32,35,38]. It was argued that the low work function of the rare earth atom forces it to transfer a charge to the half-filled 4*d_z_*^2^ orbital of the Ta atom. Thus, the MLC gains extra stability by this charge transfer as discussed also in [39]. The question then arises: how much of the rare-earth atom can be replaced by a divalent alkali earth atom, like strontium, while still retaining the stability of the MLC compound? This issue was deliberated in the case of the MLC Sr*_x_*La_1−_*_x_*S–CrS_2_ [40], Sr*_x_*La_1−_*_x_*S–VS_2_ [41–42], Ca*_x_*Bi_1−_*_x_*S–TiS_2_ [43] and Sr*_x_*La_1−_*_x_*S–NbS_2_ [44–45]. The stability limit with respect to the Sr exchange in the lattice varies from one MLC to the other. For example, in Sr*_x_*La_1−_*_x_*S–CrS_2_ the stability limit was found to be about 20 atom % [40]. In this case, the authors showed that the maximum Sr content is determined by a charge balance, i.e., the amount of La vacancies in the parent MLC compound. For smaller amounts of lanthanum atoms (i.e., larger Sr content in the MS sublattice), the charge transfer to the CrS_2_ slab becomes smaller. Therefore, the hexagonal (layered) phase of CrS_2_ becomes unstable beyond 20 atom % Sr and the MLC vanishes. While the parent compound has a full *d_z_*^2^ Cr level and is a semiconductor, the Sr-substituted compound is electron deficient, and hence, is metallic. The maximum content of Sr in Sr*_x_*La_1−_*_x_*S–NbS_2_ was found to be 45 atom %, [44–45] and 35 atom % in Sr*_x_*La_1−_*_x_*–VS_2_ [41–42]. In this latter case, the MLC can be transformed from a Mott insulator into a metallic state at a Sr content of 30 atom %.

Interestingly, in several MLCs, the *c*-axis was found to expand with increasing Sr concentration [40,45–46]. The expansion of the *c*-axis can be ascribed to the reduced charge transfer from the Sr*_x_*La_1−_*_x_*S rock-salt slab to the NbS_2_ (CrS_2_) slab, which leads to the weakening of the interlayer polar forces between the MX and the TX_2_ units. This point was discussed also in relation to the number of *f*-electrons in the rare-earth series in LnS–TaS_2_ (MLC) nanotubes [46]. Thus, the interlayer spacing (*c*-axis) was found to shrink in the electron-rich late lanthanides (Gd, Yb) compared to the early ones (La, Pr). This trend was attributed to the increase of the charge transfer from the rare-earth atom (in MX) to the Ta atom (in TX_2_) as their atomic number increases along the lanthanide series of atoms. Alternatively, the shrinking interlayer spacing can be stated as originating from the smaller ionic radius of the rare-earth atom with increasing Z-number. This trend was further confirmed by following the blue shift of the Raman *E*_2g_^1^-mode in these compounds, which was directly associated with the degree of charge transfer in the MLC nanotubes [46]. Finally, the compound SrTa_2_S_5_ with hexagonal structure, which can be possibly described as SrS–(TaS_2_)_2_ MLC, was found to exhibit a transition to a superconductor state at 3.16 K. [47]. A few authors suggested that increasing the charge transfer from the MX unit to the TX_2_ suppresses the charge density wave (CDW) transition, promoting thereby the superconducting state of the MLC [32]. This effect can be refined by controlling the Sr to rare-earth atoms in the MX lattice of the MLC.

In the present work, the synthesis and characterization of nanotubes from the series Sr*_x_*La_1−_*_x_*S–TaS_2_ with ascending Sr content was undertaken. In particular, high-resolution transmission electron microscopy and Raman spectroscopy served as the main experimental tools to analyze these new nanotubes. Density functional theory (DFT) calculations were used to study the chemical bonding and the stability of the Sr*_x_*La_1−_*_x_*–TaS_2_ misfits as a function of Sr content to unveil the origin of the morphological and structural peculiarities observed experimentally.

## Experimental Details

### Synthesis

The synthesis was carried out via the chemical vapor transport (CVT) technique following a procedure similar to the one which has been described in the literature already [17–20,37]. The precursors, La (Sigma-Aldrich Chemicals 99.5%), SrS (Sigma-Aldrich Chemical), Ta (Alfa Aesar 99.9%) and S (Sigma-Aldrich Chemical 99.99%), were taken in the molar proportion 1:1:3 (La+Sr)/Ta/S and mixed with a catalytic amount of TaCl_5_ (Sigma-Aldrich Chemicals 99.99%). The mixtures were mechanically ground under inert atmosphere in a glove box and charged into quartz ampoules. The ampoules were evacuated and sealed under a vacuum on the order of 1 × 10^−5^ Torr and placed in a preheated two-zone vertical furnace. The annealing was performed following a two-step protocol under constant monitoring of the temperature inside the furnace. In the first step the ampoule was submitted to a thermal gradient of 390 °C (bottom edge) and **≈**800 °C (upper edge). After one hour the ampoule was moved inside the furnace and exposed to an opposite temperature gradient (860 °C at the lower edge and **≈**390 °C at the upper edge). After 6 h the ampoule was withdrawn from the furnace and cooled down to room temperature in the air. Under these conditions the mass transport was negligible allowing the complete recovery of the product present at the lower edge of the ampoule. The concentration of Sr in the precursor is expressed as atom % (i.e., 100 − atom % of La). MLC Sr*_x_*La_1−_*_x_*S–TaS_2_ nanotubes with varying Sr compositions (Sr atom %: 10%, 20%, 40%, 60%, corresponding to *x* = 0.1, 0.2, 0.4 and 0.6, respectively) were prepared.

### Electron microscopy

Scanning electron microscopy (SEM) was done by LEO model Supra 55VP SEM. Transmission electron microscopy (TEM) and scanning transmission electron microscopy (STEM) were performed on a Titan Themis 80-300 microscope with probe and image spherical aberration (Cs) correctors, at 200 kV. Energy-dispersive X-ray spectroscopy (EDX) was performed using a SuperX EDX detector attached to this microscope using Bruker Esprit software. The quantification was done using the Cliff–Lorimer method. The samples for electron microscopy were prepared by dispersing the synthesized powder in ethyl alcohol, followed by ultrasonication and drying a drop of this dispersion onto a lacey-carbon-supported Cu/Ni grid. To minimize contamination during imaging, the TEM specimens were heated in a vacuum chamber at 60 °C overnight followed by 3 seconds of oxygen plasma exposure prior to the electron microscopy analysis.

### Raman spectroscopy

Raman spectroscopy measurements were recorded from 100 to 800 cm^−1^ on individual nanotubes using the reflection mode. The LabRAM HR Evolution (HORIBA, France) set-up was used for this analysis. The excitation was performed with a 633 nm laser having 2 mW maximum power. The set-up uses an 800 mm spectrograph, which allows for a high spectral resolution and low stray light. The pixel resolution is ≈1.8 cm^−1^ when working with a 600 gr/mm grating and a 633 nm laser. The sample was illuminated using a ×100 objective (MPlanFL NA 0.9, Olympus, Japan). The Raman spectra were measured using a 1024 × 256 pixel open electrode front-illuminated CCD camera cooled to −60 °C (Syncerity, HORIBA, USA). The system utilizes an open confocal microscope (Olympus BXFM) with a spatial resolution better than 1 μm. The measurements were done with the laser beam focused on a single nanotube at a time.

### DFT calculations

All calculations were performed within the framework of the density-functional theory (DFT) using the SIESTA 4.0 implementation [48–49]. The Perdew–Burke–Ernzerhof (PBE) parametrization of the exchange-correlation potential within the generalized gradient approximation (GGA) was used. The core electrons were treated, applying norm-conserving Troullier–Martins pseudopotentials. The pseudopotential core radii (given in the following in brackets, in *a*_B_ units) for the valence shells were chosen as 6s^2^(3.47)6p^0^(3.74)5d^1^(3.22) for La, 6s^1^(2.55)6p^0^(2.74)5d^4^(2.55) for Ta, 5s^2^(3.58)5p^0^(3.76)4d^0^(3.58) for Sr, and 3s^2^(1.69)3p^4^(1.69)3d^0^(1.69) for S. A double-ζ polarized basis set was employed for all elements. The *k*-point mesh was generated by the method of Monkhorst and Pack with the cutoff of 15 Å for *k*-point sampling. The real-space grid used for the numeric integrations was generated with the energy cutoff of 300 Ry. All calculations were performed using variable-cell and atomic position relaxations, with convergence criteria corresponding to the maximum residual stress of 0.1 GPa for each component of the stress tensor, and the maximum residual force component of 0.05 eV/Å. Preliminary test calculations of binary sulfides SrS, LaS_2_, La_2_S_3_, and 2*H*-TaS_2_ revealed a good suitability of the chosen approach for the description of the geometry. The difference between the experimental and computed lattice parameters is within ±2%.

## Results and Discussion

**Figure 1:**
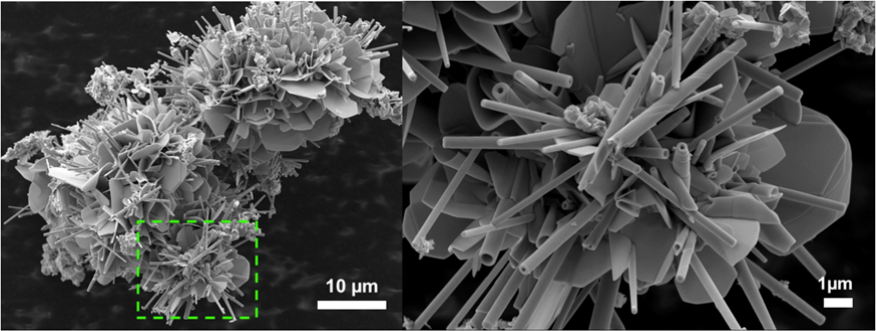
SEM images (two different magnifications) of La*_x_*Sr_1−_*_x_*S−TaS_2_ powder prepared from 10 atom % Sr (90 atom % La) in the precursor. Tubular structures and conical nanoscrolls along with sheet-like morphology are visible.

Four kinds of Sr-substituted LaS–TaS_2_ samples, i.e., Sr*_x_*La_1−_*_x_*S–TaS_2_ with increasing Sr content in the precursor (10 to 60 atom %), were prepared. Concomitantly, the corresponding content of La atoms in the precursor was reduced from 90 to 40 atom %. Here the percentage refers to the atom % in the MS precursor. SEM analysis showed that the nanotubes are indeed formed in all the different compositions, although the yield of the nanotubes decreased with increasing Sr content. Figure 1 shows an overall image of the product obtained with 10 atom % Sr (90 atom % La) in the precursor. The powder consisted of tubular and sheet like morphologies.

**Figure 2:**
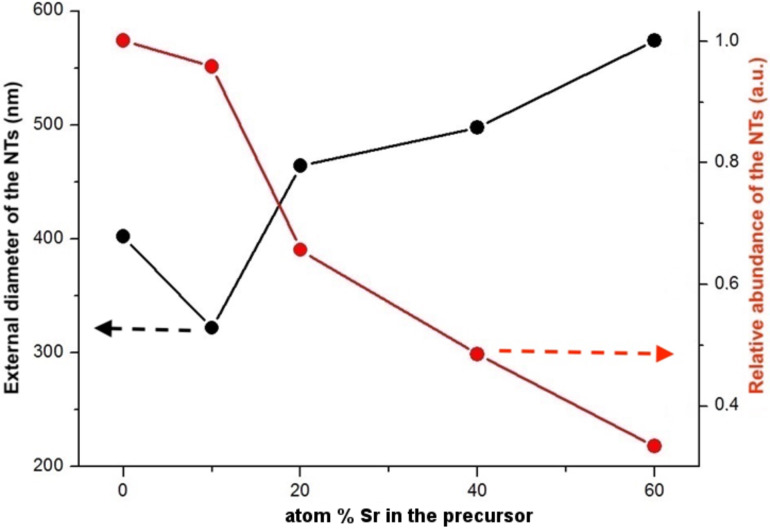
Relative abundance of the nanotubes (red curve) in the product and their average outer diameter (black curve) determined from the SEM images.

Figure 2 shows the statistical analysis of the SEM micrographs where the relative abundance of the nanotubes and their outer diameter are reported. Clearly the abundance of the nanotubes decreased with increasing Sr concentration in the precursor. Above 60 atom %, the presence of nanotubes was significantly reduced and most of the remaining material consisted of less well-defined MLC platelets. Considering that the nanotubes make up about 30–40% of the product in the pure LaS–TaS_2_ [35], it is clear from the graph (red curve) that the nanotubes form a minority phase (5% of the product) already at 40 atom % Sr in the precursor. The reduced abundance of the NTs with increasing Sr content in the precursor may be rationalized on the basis of the reduced charge transfer between the MS unit (La*_x_*Sr_1−_*_x_*S) and the TaS_2_ layer in the MLC which destabilize the MLC lattice. The increased (average) external diameter of the nanotubes with increasing Sr content in the precursor could be also attributed to the reduced charge transfer between the MS and TaS_2_ sublattices, which weakens the polar interaction in the lattice and leads to expansion of the interlayer spacing and the overall radius of the nanotubes. However, this is only one plausible explanation and more careful study of this tendency is undertaken by means of DFT calculations vide infra.

**Figure 3:**
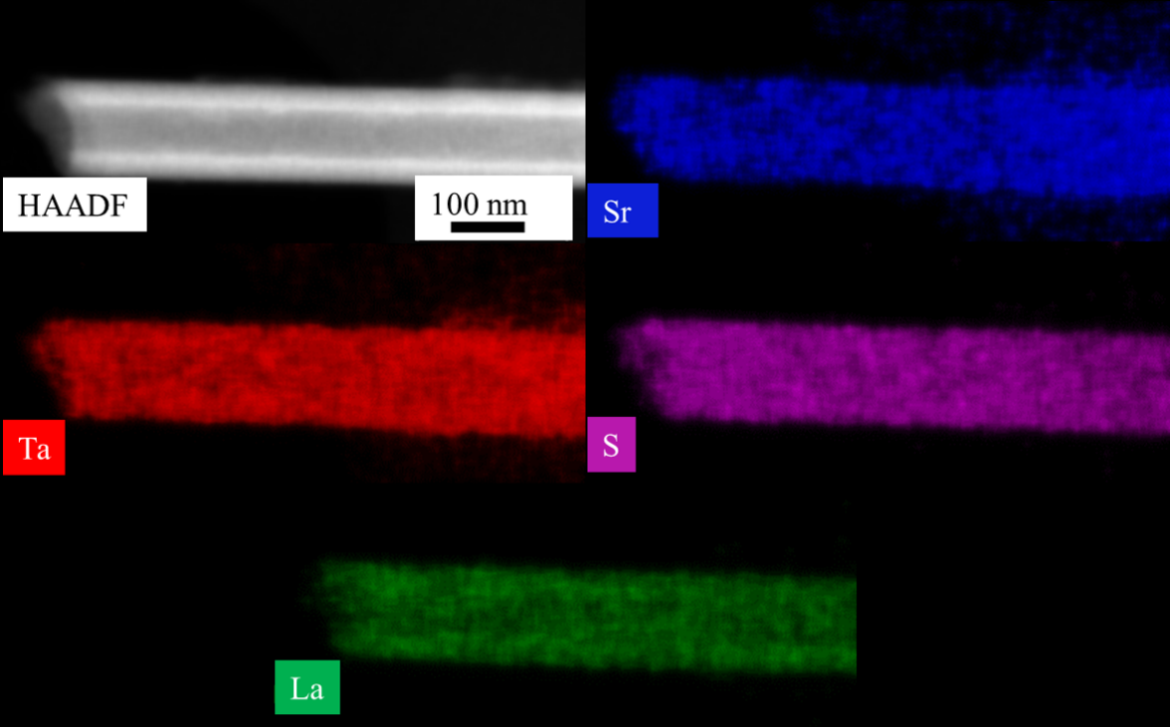
HAADF-STEM image (top left) and EDX elemental mapping of Sr, Ta, S, and La in a Sr*_x_*La_1−_*_x_*S–TaS_2_ nanotube prepared from 10 atom % of Sr in the precursor.

The tubular morphology of the Sr*_x_*La_1−_*_x_*S–TaS_2_ sample with 10 atom % Sr in the precursor was further confirmed by high-angle annular dark field (HAADF)-STEM analysis as shown in Figure 3 (top left). The brighter walls and the darker hollow region are typical of a tubular morphology. The compositional map obtained from the nanotube (Figure 3 (top right)) shows that Sr is uniformly distributed in the nanotube. Elemental quantification from the EDX spectra indicates that 7–11 atom % of Sr substitution (93–89 atom % La) is achieved in a nanotube. The overlap of the L and Cu K lines for Ta might induce some error in quantification in the case of Ta. Also, due to the overlap of the L line for Sr and the M line for Ta, the weak Sr K line is used for mapping and quantification of Sr. Nanotubes with a wide range of inner and outer diameters were observed; inner diameters of 20–70 nm and outer diameters of 80–300 nm were found.

In the case of the LaS–TaS_2_ misfit-layered compound, the structure consists of alternating slabs of LaS and TaS_2_ with different crystallographic structures. LaS adopts a distorted NaCl structure and the TaS_2_ can be indexed in a pseudo-hexagonal unit cell [35,38].

**Figure 4:**
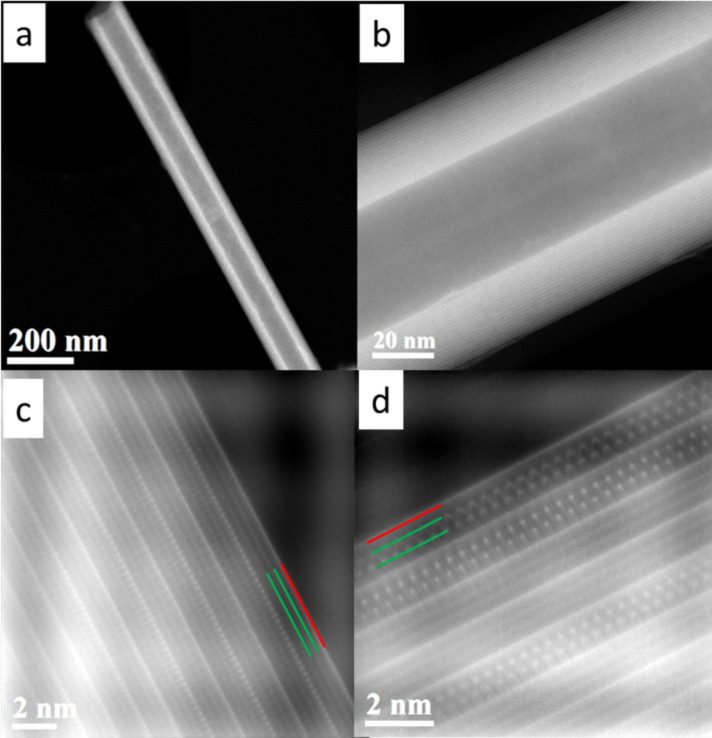
(a), (b) HAADF STEM images of Sr*_x_*La_1-_*_x_*S-TaS_2_ (Sr 10 atom %) nanotubes. (c) High-resolution image of (a) indicating that alternating TaS_2_ layers (red line) are of two different orientations. (d) High-resolution image of (b) showing the Sr*_x_*La_1-_*_x_*S double layer clearly (two atomic planes indicated by green lines).

Figure 4 shows low-magnification and high-resolution HAADF-STEM images of two LaS–TaS_2_ (10 atom % Sr in the precursor) nanotubes. One can see the stacking of the Sr*_x_*La_1−_*_x_*S (indicated by green lines) and TaS_2_ (indicated by red line) layers in Figure 4c and that TaS_2_ appears brighter due to the Z dependence of contrast [50], as compared to the Sr*_x_*La_1−_*_x_*S double layer between them. From Figure 4c, one can also conclude that the alternating TaS_2_ layers have different atomic arrangements, indicating the presence of two folding vectors for TaS_2_. The atomic arrangement of the Sr*_x_*La_1−_*_x_*S double layers are better revealed in the nanotube shown in Figure 4d.

**Figure 5:**
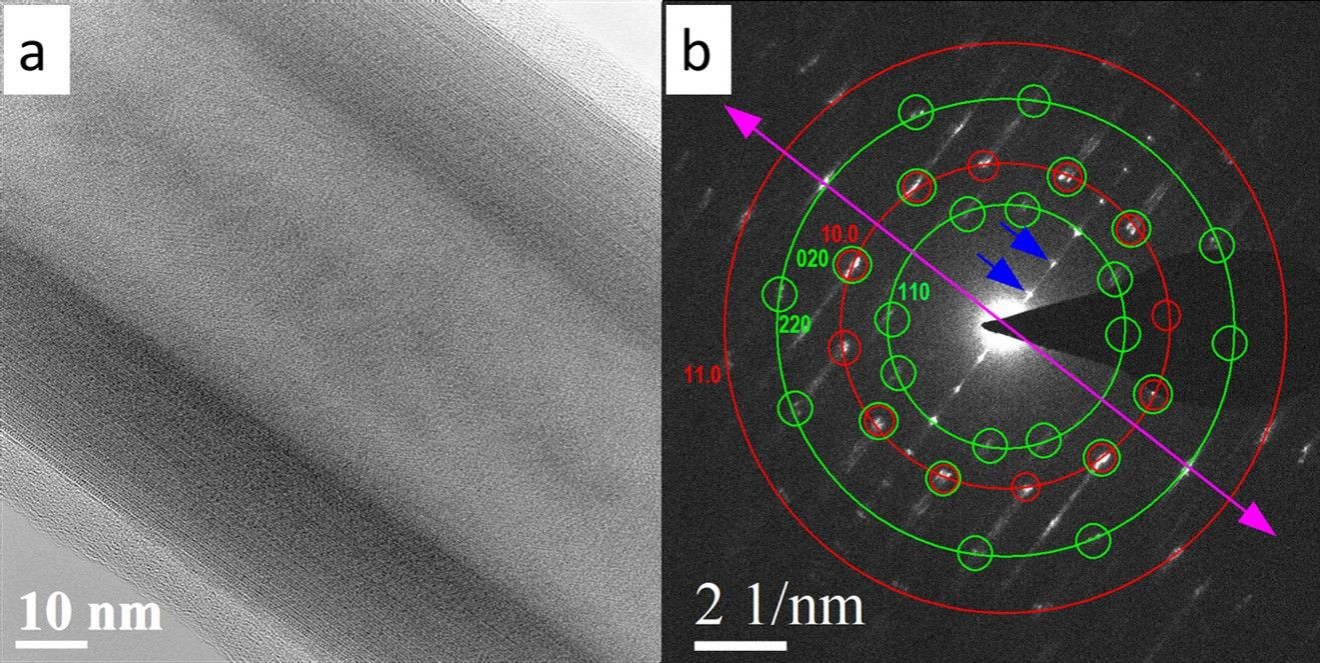
a) HRTEM image of a Sr*_x_*La_1−_*_x_*S–TaS_2_ (10 atom % Sr in the precursor) nanotube showing 1.157 nm periodicity along the *c*-axis. b) Selected area electron diffraction showing the orientation relationship between LaS layers (green) and TaS_2_ layers (red). The nanotube axis is shown as a pink arrow and the basal reflections are marked with blue arrows.

The structure of this nanotube was further analyzed by a selected area electron diffraction (SAED) pattern as shown in Figure 5. Twelve pairs of spots corresponding to (10.0) planes with a *d* spacing of 2.82 Å and 12 pairs of spots corresponding to (11.0) planes (marked on the red circles) with a spacing of 1.63 Å were observed for TaS_2_. The multiplicity of these planes is 6, therefore we can confirm the presence of two folding vectors for TaS_2_ in the nanotube investigated. Eight pairs of spots of 3.97 Å and 2.04 Å were also observed (marked on the green circles), which could be indexed to (110) and (220), respectively, of LaS. The multiplicity factor of these planes is four, therefore the presence of two folding vectors can be confirmed for the LaS lattice as well. The splitting of the spots indicates the chiral nature of the nanotube. The chiral angles calculated from the splitting of the spots, *hk*.0 of LaS and TaS_2_, were ≤3°. Also, it was observed that the (020) spots of LaS coincide with the (10.0) of TaS_2_ even though they are not parallel to the nanotube axis, marked as a pink arrow. This is in contrast to the previously reported tubular LnS–TaS_2_ (Ln = rare earth) material, where the majority of nanotubes analyzed were of one common *b*-axis parallel to the nanotube axis [35]. The basal reflections are the spots appearing perpendicular to the nanotube axis. From the HRTEM images, a periodicity of **≈**1.16 nm along the *c-*axis was concluded (Figure 5a).

**Figure 6:**
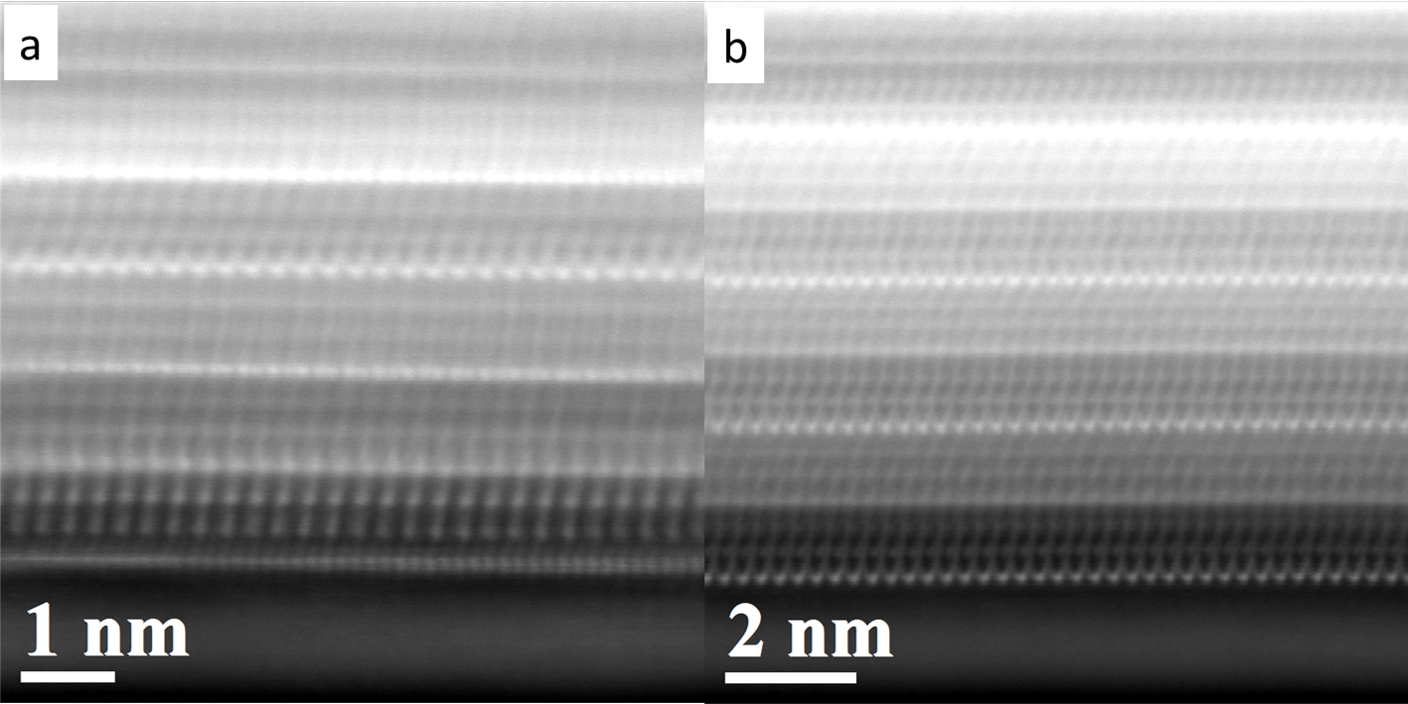
(a,b) HAADF-STEM images of a Sr*_x_*La_1−_*_x_*S–TaS_2_ (20 atom % Sr in the precursor) nanotube showing multiple orientations of TaS_2_ layers.

**Figure 7:**
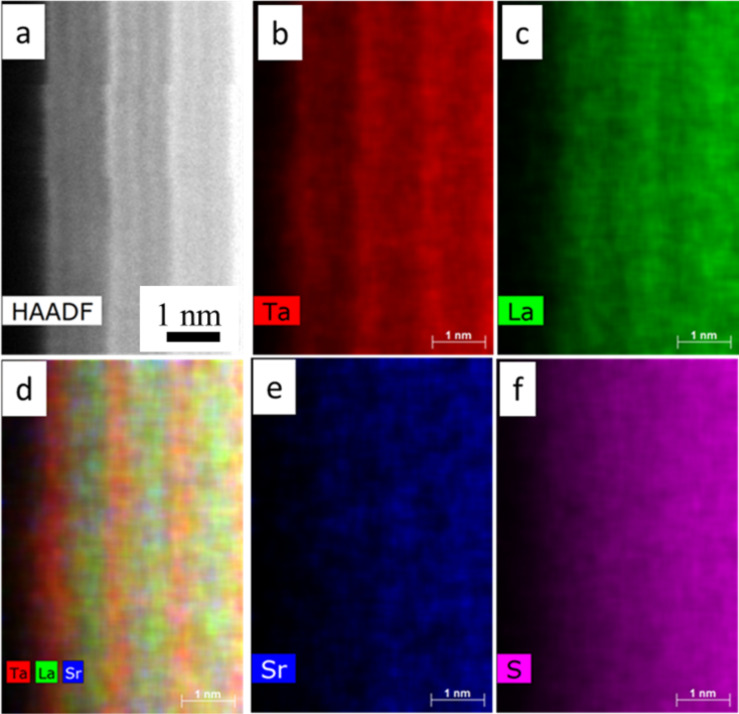
(a) HAADF-STEM image and (b–f) EDX elemental mapping on a Sr*_x_*La_1−_*_x_*S–TaS_2_ (40 atom % Sr in the precursor) nanotube of (b) Ta, (c) La, (d) a composite map of the elements, (e) Sr and (f) S.

The analysis of HAADF-STEM images of a Sr*_x_*La_1−_*_x_*S–TaS_2_ nanotube from a sample containing 20 atom % Sr substitution shows that nanotubes with different folding vectors are present. For example, in Figure 6a, the TaS_2_ layer has multiple orientations whereas in Figure 6b, it has two different orientations. EDX quantification indicates that 25–37 atom % of Sr/La substitution was achieved in the nanotubes, i.e., the rock-salt lattice contained 70 atom % La and ≈30 atom % Sr. From the HAADF-STEM image in Figure 7, the TaS_2_ layer with higher HAADF intensity can be identified as well as the LaS double layer in between them.

EDX elemental maps on the LaS–TaS_2_ sample with 40 atom % Sr substitution (Figure 7) show that the Sr map as well as the La map matches with the LaS double layer position and the Ta map shows higher intensity at the TaS_2_ layer, as expected from the HAADF intensity. To clearly see this, the map data with Ta, La and Sr are merged in Figure 7d. This indicates that the Sr substitutes for La (Sr_La_) in the crystal. EDX quantification showed that 28–41 atom % of Sr substitution of the La site was obtained in the analyzed nanotubes. The S map shows more or less uniform distribution in the nanotube.

HRTEM imaging and SAED analysis were carried out on a Sr*_x_*La_1−_*_x_*S–TaS_2_ sample with 60 atom % Sr in the precursor (40 atom % La). The nanotube was found to have an interlayer periodicity of **≈**1.18 nm along the *c-*axis (Figure S1a, Supporting Information File 1). The interlayer spacing is larger for the tube with 60 atom % Sr in the precursor compared with that of the 10 atom % (1.16 nm). This result reflects the weaker interlayer interaction in the nanotube, i.e., the reduced charge transfer with increasing Sr concentration. From SAED (Figure S1b, Supporting Information File 1), two folding vectors of Sr*_x_*La_1−_*_x_*S and TaS_2_ were observed in the nanotube. Also, it was observed that the *b*-axis (020) of LaS/(10.0) of TaS_2_ is not parallel to the nanotube axis, which is analogous to the situation in the Sr*_x_*La_1−_*_x_*S–TaS_2_ nanotube with 10 atom % Sr in the precursor (Figure 5). However, we have come across nanotubes with different orientations of TaS_2_ and Sr*_x_*La_1−_*_x_*S layers in the sample.

**Figure 8:**
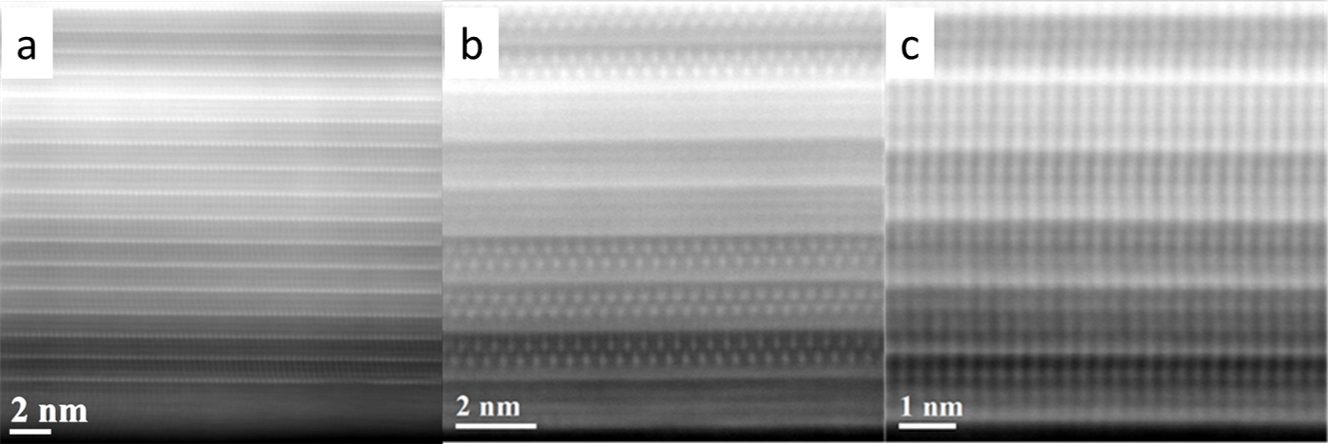
(a–c). HAADF-STEM images of a Sr*_x_*La_1−_*_x_*S–TaS_2_ (with Sr 60 atom % in the precursor) nanotube showing multiple orientation of TaS_2_ layers and Sr*_x_*La_1−_*_x_*S double layers.

In the HAADF-STEM image in Figure 8, three nanotubes with three different arrangements of constituent layers are found. A TaS_2_ layer with higher HAADF intensity can be identified as well as the LaS double layer in between them. Elemental EDX maps of the Sr*_x_*La_1−_*_x_*S–TaS_2_ sample with 60 atom % Sr substitution (Figure S2, Supporting Information File 1) show that the Sr map as well as the La map matches with the position of the double layer rock-salt lattice, whereas the Ta map shows higher intensity at the TaS_2_ layer, as expected from the HAADF intensity image. For better understanding, the map data with Ta, La and Sr are merged in Figure S2d, Supporting Information File 1. This indicates that the Sr atoms substitute for La in the lattice. EDX quantification indicated that the La/Sr ratio is in the range 38–61 (La):62–39 (Sr) in the analyzed nanotubes. This analysis shows that the Sr atoms can substitute for the La atoms up to about 60 atom %. The HRTEM/EDX analysis does not indicate any Sr substitution into the TaS_2_ lattice (the detection limit is about 0.5 atom %). Presumably, above that Sr concentration, the charge balance is lost, and the MLC nanotubes become unstable. The diameter distribution obtained from the TEM/STEM images is shown in Table S1, Supporting Information File 1. Samples with 40% and 60% have larger diameters than samples with 10% and 20%. There is no linear increase as the order is reversed between 40% and 60%.

**Figure 9:**
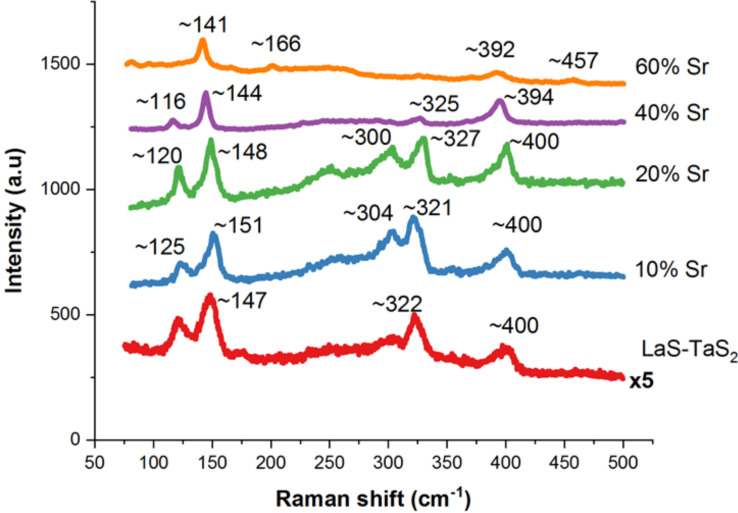
Raman spectra of Sr*_x_*La_1−_*_x_*S–TaS_2_ nanotubes with different Sr content. Additionally, the reference Raman spectrum is shown for LaS–TaS_2_.

The Sr*_x_*La_1−_*_x_*S–TaS_2_ nanotubes with different Sr content were also analyzed by Raman spectroscopy and Figure 9 summarizes this analysis. The Raman spectra of MLC were analyzed first by Kisoda et al. [51], and were further elaborated in [32] and [43]. The range between 100–150 cm^−1^ was assigned to the intralayer vibrations of the LnS lattice (Ln = rare earth). The high energy range 250–400 cm^−1^ was associated with the intralayer vibrations of the hexagonal TaS_2_ lattice. The peak at 253 cm^−1^ was assigned to the two-phonon bands of the TaS_2_ slab. The 125 cm^−1^ peak was assigned to the out-of-phase vibration of the LaS lattice. On the other hand, the 150 cm^−1^ peak was assigned to the in-phase phonon of LaS. The *E*_2g_ mode of the TaS_2_ in the MLC lattice is found in the energy range of about 321–325 cm^−1^. This peak is blue-shifted compared to the pure TaS_2_ (279 cm^−1^), which is attributed to the characteristic charge transfer from the LaS slab to that of TaS_2_. The broad band between **≈**240 and 304 cm^−1^ is attributed to the two-phonon band [52], whereas the 400 cm^−1^ transition was assigned to the A_1g_ vibration of the 2*H*-TaS_2_. Obviously, the diameter, number of layers and chirality varies from one nanotube to the next, and from one batch to the next. This polydispersity leads to the broadening of the peaks and minor shifts in their positions. Increasing the Sr content in the lattice of the rock-salt slab in place of the La has several ramifications on the Raman spectrum. First, the Sr atom is lighter (87.6 au) than the La atom (138.9 au), which should lead to an increase in the frequency of the oscillating atoms. However, at the same time, Sr bears a smaller charge (+2 instead of the +3 for the La). In the absence of any accurate calculation, one can nevertheless conclude that the charge transfer between the La*_x_*Sr_1−_*_x_*S slab to the TaS_2_ one is reduced with increasing Sr content in the lattice. Therefore, the polar forces between the two slabs are weaker and the interlayer spacing becomes larger, which is expected to lead to softening of the Raman modes (i.e., lower wavenumbers). Furthermore, assuming the Sr distribution in the lattice is random, the structural fluctuations increase, leading also to broadening of the modes. Notwithstanding these variations, some systematic changes in the Raman spectra with Sr content in the lattice are noticeable. Most importantly, a clear softening in the Raman modes is observed, which suggests that the predominant effect of the Sr substitution is to reduce the strain in the lattice due to the reduced charge transfer between the layers, and consequently, the lattice strain. It also appears that beyond an Sr content of **≈**60 atom %, the MLC lattice becomes unstable and new modes >680 cm^−1^ appear, which is typical for metal oxides.

**Figure 10:**
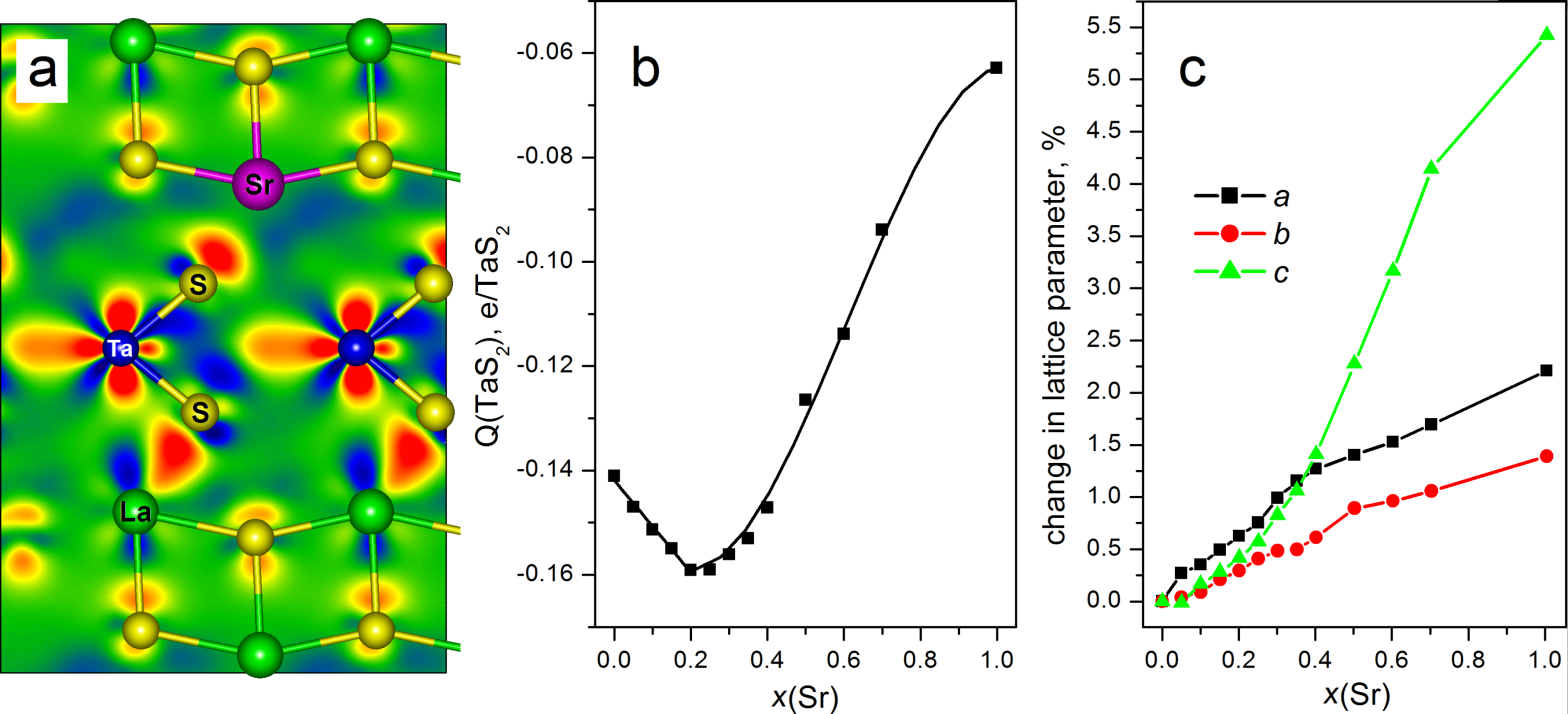
DFT calculations presenting the peculiarities of the chemical bonding within the Sr*_x_*La_1−_*_x_*S–TaS_2_ misfits, as a function of the Sr content. (a) Charge redistribution map at the interface formed between the Sr_0.05_La_0.95_S slab and single TaS_2_ layer. Red and blue colors on the map correspond to an increase and decrease of electron density on 0.05 e/Å^3^, respectively, and the green color corresponds to no change when compared to isolated layers. (b) The value of charge transfer (*Q*) from the Sr*_x_*La_1−_*_x_*S to TaS_2_ layer, and (c) modulation of the lattice parameters (*a*, *b*, *c*) with ascending Sr content (and reduced La content).

DFT calculations of the pristine LaS–TaS_2_ and the Sr*_x_*La_1−_*_x_*S–TaS_2_ MLC bulk alloys were undertaken. Overall, the trends observed in the morphology and in the lattice parameters of MLC upon Sr alloying were confirmed by the results of the DFT calculations. As a prototypic model structure, the approximant (LaS)_1.11_TaS_2_ was chosen, whose supercell includes one LaS and one TaS_2_ layer (20 LaS and 18 TaS_2_ units). The evolution of the electronic structure and the lattice parameters of Sr*_x_*La_1−_*_x_*S–TaS_2_ was traced by consecutive exchange of La atoms with Sr atoms, keeping the Sr atoms as far from each other as possible and preventing their clustering together. The in-plane lattice parameters of the pristine compound are found using DFT calculations as *a* = 2.94 nm and *b* = 0.58 nm, while the interlayer LaS–TaS_2_ spacing along the *c*-axis is equal to 1.15 nm. The relative thermodynamic stability of (Sr*_x_*La_1−_*_x_*S)_1.11_TaS_2_ misfits was estimated using the formation energy Δ*E* for the model reaction (LaS)_20_(TaS_2_)_18_ + *x*SrS → (Sr*_x_*La_1−_*_x_*S)_20_(TaS_2_)_18_ + *x*LaS, where LaS refers to the hypothetical fcc compound. The DFT calculations confirmed that the consecutive substitution of La with Sr leads to a gradual diminution in the stability and to an expansion in the Sr*_x_*La_1−_*_x_*–TaS_2_ lattice along all crystallographic directions (Figure 10 and Figure S3 in Supporting Information File 1). Meanwhile, a closer inspection of the modulations in the functions of Δ*E*, *a*, *b* and *c* has disclosed inflection points near 30–40 atom % Sr content. Such functional behavior points to a sudden change of an intrinsic property of the lattice. Indeed, a qualitative change in the chemical bonding between Sr*_x_*La_1−_*_x_*S and TaS_2_ parts of the lattice has been registered, while the MLC lattice was found to preserve its integrity after the geometry optimization. The intuitive charge transfer from the electron-rich LaS layer to the electrophilic TaS_2_ layer demonstrates a non-monotonic behavior upon Sr doping of the LaS part. In fact, the charge transfer per TaS_2_-unit, *Q*, first slightly increases in absolute value with increasing Sr content in the rock-salt lattice from 0.14 to 0.16 e^−^ per TaS_2_ unit and then reaches a minimum (maximum in absolute value) at 20 atom % Sr content (Figure 10a). At higher Sr content, the function *Q* displays an S-shaped profile and diminishes (in absolute value) dramatically from 0.16 to 0.06 e^−^/TaS_2_ of a hypothetical (SrS)_1.11_TaS_2_ misfit (i.e., upon a complete La to Sr substitution). This phenomenon can be explained by the excessive electron-donating ability of the LaS layer. Even in the presuming case of the single electron transfer from La^2+^ to Ta^5+^, the number of La atoms within the (LaS)_1.11_TaS_2_ misfit remains too excessive for the number of Ta atoms. Several options can be suggested for accommodation of excessive electrons within the MLC lattice. An analysis of the electron density distribution within the studied (Sr*_x_*La_1−_*_x_*S)_1.11_TaS_2_ misfits unveils the localization of excessive electron density due to charge transfer not only at 5d*_z_*^2^ orbitals of Ta atoms. Rather, redistribution and alignment of the charge between the La atoms and the S atoms of TaS_2_ layer can be also observed (see the red regions between La and S in Figure 10a). Such localized and unidirectional enhancement of the electron density can be ascribed to the coordination (covalent-like) La–S(TaS_2_) bonding in the MLC. Noticeably, the substituting Sr atoms do not participate in the formation of any bonding within the TaS_2_ layer (represented by the green color in Figure 10a). The electron density in the vicinity of the Sr atoms (in the Sr*_x_*La_1−_*_x_*S lattice) remains essentially unperturbed compared to the corresponding free-standing SrS part. Quantitatively, the degree of bond covalency can be also discussed using the crystal orbital overlap populations (COOPs) between atoms. For example, the La–S bonds within LaS part of the (Sr_0.05_La_0.95_S)_1.11_TaS_2_ misfit are characterized by COOPs in the order of 0.2 e^−^, while the COOPs for Sr–S bonds within the Sr*_x_*La_1−_*_x_*S lattice are equal to 0.10 e^−^. Depending on the positional coincidence between incommensurate LaS and TaS_2_ layers, the La atoms can form 1–3 coordinate bonds with the S atoms of the TaS_2_, where the COOPs are equal to 0.1–0.2 e^−^. In contrast to this, the COOP for the bonding between Sr and the S atoms of TaS_2_ does not exceed 0.05 e^−^ and becomes even smaller in misfits with a higher Sr content. As expected, the calculations confirm the presumably ionic character of the Sr–S interaction and a covalent-like La–S interaction between the atoms of different units of the (Sr*_x_*La_1−_*_x_*S)_1.11_TaS_2_ misfits. Obviously, the charge transfer from LaS to the 5d*_z_*^2^ orbitals of Ta atoms is only slightly perturbed by the interfacial coordination between the La atom of LaS and the S atom of the TaS_2_ slab. The gradual removal of excessive electrons from the LaS upon Sr alloying allows one to observe the maximal charge transfer (minimal value *Q*) up to the ratio La/Ta 8:9 (20 atom % Sr). Since the Sr does not contribute to the charge transfer between the two slabs, this small increase in charge transfer with increasing Sr content can be possibly attributed to a purely geometric effect. The Sr diameter is 132 pm while La is 117 pm, and hence the lattice is compressed upon substitution of La by Sr atom. Any further Sr insertion leads to electron deficiency within the Sr*_x_*La_1−_*_x_*S–TaS_2_ misfits and to the weakening of cohesion between LaS and TaS_2_ layers. The electronic density-of-states (DOSs) for the misfits with a Sr content up to 20% are quite similar to the pristine compound (Figure S4, Supporting Information File 1). A minor difference between 0 and 20 atom % Sr can be noticed in the shift of the S3*p*-states of the TaS_2_ part with energies −5…−6 eV towards *E*_F_ (see Figure S4, Supporting Information File 1) at ascending Sr content. At a Sr content of about 40 atom % the DOS difference with respect to the DOS of pristine LaS–TaS_2_ MLC becomes more pronounced: a clear shoulder of the valence band appears at −1.0...1.5 eV (see Figure S4, Supporting Information File 1). It consists of S3*p*-states, which are responsible for the Sr–S bonding within the SrS part. At this Sr content (40 atom %) the slope of the curves for all three lattice parameters varies substantially (Figure 10c and Figure S1c in Supporting Information File 1). Most prominently, the rate of change of the interlayer distance (*c*-axis) attains a steeper slope, than for the *a* or *b* lattice parameters (Figure 10c). Such escalation of the interlayer distance correlates with the occurrence of endothermic formation energies Δ*E* (Figure S3, Supporting Information File 1). In addition, the loss in stability can be more remarkable due to an uneven distribution of Sr atoms. Particularly, the (Sr_0.50_La_0.50_S)_1.11_TaS_2_ misfit with strictly separated SrS and LaS fragments is less stable, than the isostoichiometric misfit with an even Sr distribution in the LaS slab, by a mere 0.08 eV/TaS_2_.

An alternative arrangement of the MLC to sustain excess charge transfer from the rock-salt sublattice is to form a pair of TaS_2_ layers for each rock-salt layer. Particularly, the rise of a stacking fault like a supernumerary TaS_2_ layer within the "planar" LaS–TaS_2_ misfit is highly likely. However, this arrangement could not be confirmed experimentally by the detailed TEM analysis of the samples and hence it can be concluded that this mechanism is not relevant to the present study. A curved morphology may also effectively accommodate the excessive electrons, e.g., sinusoidal-like TaS_2_ layers between planar LaS slabs or sinusoidal-like alteration of both TaS_2_ and LaS layers with the TaS_2_ layer being on the outer surface (convex) while the LaS layer is on the inner part of the sinusoidal surface (concave). Since the TaS_2_ is the outermost (convex) layer, the number of acceptor units (TaS_2_) is effectively enhanced as compared to the number of donor units in inner (convex) LaS slab. Finally, another example includes nanotubes or nanoscrolls consisting of both TaS_2_ layers and LaS slabs, where the TaS_2_ unit would tend to form the external side of the walls. Indeed, the TEM analysis confirms that the TaS_2_ is the outermost layer in these nanotubes and hence this is the likely mechanism to compensate for excess electrons from molecular-like slabs (LaS) in the nanotube. Therefore, the Sr alloying and the nanotubular morphology act similarly in regulating the charge imbalance, i.e., in charge transfer and interfacial bonding within the LaS–TaS_2_ misfits. Therefore, progressive Sr doping should disqualify the necessity of nanotubular morphology. Not surprisingly, the interlayer distance (*c*-axis) and the overall diameter of the Sr*_x_*La_1−_*_x_*S–TaS_2_ misfit nanotubes increases with higher Sr content in the lattice.

## Conclusion

In conclusion, new alloys of misfit-layered compounds with strontium atoms substituting for the La atoms in the LaS–TaS_2_ lattice, in both flake and tubular forms, were synthesized. Careful characterization with high-resolution electron microscopy and related techniques and Raman spectroscopy were carried out on the nanotubes. Clearly, the Sr atoms were found to be confined in the LaS distorted rock-salt structure. Although the Sr concentration varied from one nanotube to the next (and even within the nanotube itself), they were found to be unstable beyond ≈60 atom % Sr (40 atom % La). This phenomenon was attributed to the reduced charge transfer between the LaS slab and the TaS_2_ layer, resulting in a weakening of the interlayer polar forces. For this reason, the interlayer spacing (along the *c*-axis) was found to increase with increasing Sr content. In general, increasing the Sr content in the nanotube led to softening of the Raman modes, which is also attributed to the relaxation of the interlayer forces. DFT calculations of the approximant (Sr*_x_*La_1−_*_x_*S)_1.11_TaS_2_ showed that the amount of charge transfer from the rock-salt Sr*_x_*La_1−_*_x_*S lattice to the hexagonal TaS_2_ lattice goes through a shallow minimum at 20 atom % Sr substitution. Furthermore, the lattice of the alloy is stable up to 40 atom % Sr content, where the charge distribution and the lattice parameters thereafter exhibit abrupt changes, which can be attributed to lattice instability. The interlayer distance (along the *c*-axis) increases and the degree of charge transfer from the rock-salt to the hexagonal lattice is reduced upon increasing Sr_La_ substitution. An analysis of the charge density distribution in the Sr*_x_*La_1−_*_x_*S–TaS_2_ misfits confirms the fairly ionic nature of the Sr atoms (like that in SrS compounds), while the La atoms establish covalent-like bonding with neighbor S atoms from both Sr*_x_*La_1−_*_x_*S and TaS_2_ parts of the MLC.

## Supporting Information

File 1 Additional experimental data and calculations.

## References

[1] Tenne R, Margulis L, Genut M, Hodes G (1992). Nature.

[2] Tenne R (2006). Nat Nanotechnol.

[3] Muhr H-J, Krumeich F, Schönholzer U P, Bieri F, Niederberger M, Gauckler L J, Nesper R (2000). Adv Mater (Weinheim, Ger).

[4] Brontvein O, Stroppa D G, Popovitz-Biro R, Albu-Yaron A, Levy M, Feuerman D, Houben L, Tenne R, Gordon J M (2012). J Am Chem Soc.

[5] Brorson M, Hansen T W, Jacobsen C J H (2002). J Am Chem Soc.

[6] Brontvein O, Albu-Yaron A, Levy M, Feuerman D, Popovitz-Biro R, Tenne R, Enyashin A, Gordon J M (2015). ACS Nano.

[7] Zak A, Sallacan-Ecker L, Margolin A, Feldman Y, Popovitz-Biro R, Albu-Yaron A, Genut M, Tenne R (2010). Fullerenes, Nanotubes, Carbon Nanostruct.

[8] Therese H A, Li J, Kolb U, Tremel W (2005). Solid State Sci.

[9] Remškar M, Mrzel A (2003). Vacuum.

[10] Remskar M, Mrzel A, Virsek M, Godec M, Krause M, Kolitsch A, Singh A, Seabaugh A (2010). Nanoscale Res Lett.

[11] Levi R, Bitton O, Leitus G, Tenne R, Joselevich E (2013). Nano Lett.

[12] Fathipour S, Remskar M, Varlec A, Ajoy A, Yan R, Vishwanath S, Rouvimov S, Hwang W S, Xing H G, Jena D (2015). Appl Phys Lett.

[13] Divon Y, Levi R, Garel J, Golberg D, Tenne R, Ya’akobovitz A, Joselevich E (2017). Nano Lett.

[14] Zhang Y J, Onga M, Qin F, Shi W, Zak A, Tenne R, Smet J, Iwasa Y (2018). 2D Mater.

[15] Qin F, Shi W, Ideue T, Yoshida M, Zak A, Tenne R, Kikitsu T, Inoue D, Hashizume D, Iwasa Y (2017). Nat Commun.

[16] Qin F, Ideue T, Shi W, Zhang X-X, Yoshida M, Zak A, Tenne R, Kikitsu T, Inoue D, Hashizume D (2018). Nano Lett.

[17] Hong S Y, Popovitz-Biro R, Prior Y, Tenne R (2003). J Am Chem Soc.

[18] Radovsky G, Popovitz-Biro R, Staiger M, Gartsman K, Thomsen C, Lorenz T, Seifert G, Tenne R (2011). Angew Chem, Int Ed.

[19] Radovsky G, Popovitz-Biro R, Stroppa D G, Houben L, Tenne R (2014). Acc Chem Res.

[20] Panchakarla L S, Radovsky G, Houben L, Popovitz-Biro R, Dunin-Borkowski R E, Tenne R (2014). J Phys Chem Lett.

[21] Makovicky E, Hyde B G (1981). Non-commensurate (misfit) layer structures. Inorganic Chemistry.

[22] Wiegers G A (1996). Prog Solid State Chem.

[23] Rouxel J, Meerschaut A, Wiegers G A (1995). J Alloys Compd.

[24] Bernaerts D, Amelinckx S, Van Tendeloo G, Van Landuyt J (1997). J Cryst Growth.

[25] van Smaalen S (1992). Mater Sci Forum.

[26] Oosawa Y, Gotoh Y, Akimoto J, Tsunoda T, Sohma M, Onoda M (1992). Jpn J Appl Phys.

[27] Rouxel J, Moeelo Y, Lafond A, DiSalvo F J, Meerschaut A, Roesky R (1994). Inorg Chem.

[28] Gómez-Herrero A, Landa-Cánovas A R, Hansen S, Otero-Díaz L C (2000). Micron.

[29] Lorenz T, Joswig J-O, Seifert G (2014). Beilstein J Nanotechnol.

[30] Ohno Y (1991). Solid State Commun.

[31] Kars M, Fredrickson D C, Gómez-Herrero A, Lidin S, Rebbah A, Otero-Diáz L C (2010). Mater Res Bull.

[32] Suzuki K, Enoki T, Imaeda K (1991). Solid State Commun.

[33] Kato K, Kawada I, Takahashi T (1977). Acta Crystallogr, Sect B: Struct Crystallogr Cryst Chem.

[34] Engelsman F M R, Wiegers G A, Jellinek F, Van Laar B (1973). J Solid State Chem.

[35] Radovsky G, Popovitz-Biro R, Lorenz T, Joswig J-O, Seifert G, Houben L, Dunin-Borkowski R E, Tenne R (2016). J Mater Chem C.

[36] Lajaunie L, Radovsky G, Tenne R, Arenal R (2018). Inorg Chem.

[37] Stolovas D, Serra M, Popovitz-Biro R, Pinkas I, Houben L, Calvino J J, Joselevich E, Tenne R, Arenal R, Lajaunie L (2018). Chem Mater.

[38] Wiegers G A (1995). J Alloys Compd.

[39] Lorenz T, Baburin I A, Joswig J-O, Seifert G (2017). Isr J Chem.

[40] Cario L, Johrendt D, Lafond A, Felser C, Meerschaut A, Rouxel J (1997). Phys Rev B.

[41] Yasui Y, Nishikawa T, Kobayashi Y, Sato M, Nishioka T, Kontani M (1995). J Phys Soc Jpn.

[42] Nishikawa T, Yasui Y, Kobayashi Y, Sato M (1996). Phys C (Amsterdam, Neth).

[43] Hung Y C, Hwu S J (1993). Inorg Chem.

[44] Grippa A Y, Lidin S, D’yachenko O G, Rupasov D P, Antipov E V (2005). Mater Res Bull.

[45] D’Yachenko O G, Grippa A Y, Lidin S, Rupasov D P, Antipov E V (2004). Ferroelectrics.

[46] Serra M, Stolovas D, Houben L, Popovitz-Biro R, Pinkas I, Kampmann F, Maultzsch J, Joselevich E, Tenne R (2018). Chem – Eur J.

[47] Kijima N, Morie K, Nagata S, Shimono I (1996). J Low Temp Phys.

[48] Ordejón P, Artacho E, Soler J M (1996). Phys Rev B.

[49] Soler J M, Artacho E, Gale J D, García A, Junquera J, Ordejón P, Sánchez-Portal D (2002). J Phys: Condens Matter.

[50] Anumol E A, Enyashin A N, Batra N M, Costa P M F J, Deepak F L (2016). Nanoscale.

[51] Kisoda K, Hangyo M, Nakashima S, Suzuki K, Enoki T, Ohno Y (1995). J Phys: Condens Matter.

[52] Staiger M, Bačić V, Gillen R, Radovsky G, Gartsman K, Tenne R, Heine T, Maultzsch J, Thomsen C (2016). Phys Rev B.

