# Supplementary material for: Synthesis and characterization of quaternary La(Sr)S–TaS2 misfit-layered nanotubes
Source: Beilstein J Nanotechnol. 2019 May 24;10:1112–24. doi: 10.3762/bjnano.10.111 (PMC6541319; doi:10.3762/bjnano.10.111)
Supplement: File 1 — Additional experimental data and calculations. [file Beilstein_J_Nanotechnol-10-1112-s001.pdf]

BEILSTEIN JOURNAL OF NANOTECHNOLOGY

Supporting Information

for

Synthesis and characterization of quaternary La(Sr)S–TaS_2 misfit-layered nanotubes

Marco Serra, Erumpukuthickal Ashokkumar Anumol, Dalit Stolovas, Iddo Pinkas, Ernesto Joselevich, Reshef Tenne, Andrey Enyashin and Francis Leonard Deepak

Beilstein J. Nanotechnol. **2019,** *10,* 1112–1124. doi:10.3762/bjnano.10.111

Additional experimental data and calculations

License and Terms: This is a supporting information file under the terms of the Creative Commons Attribution License (http://creativecommons.org/licenses/by/4.0). Please note that the reuse, redistribution and reproduction in particular requires that the authors and source are credited.
The license is subject to the *Beilstein Journal of Nanotechnology* terms and conditions: (https://www.beilstein-journals.org/bjnano)

Table S1. Diameter distribution of the tubes

Sample	Average Inner Diameter (nm)	Average Outer diameter (nm)	Number of Tubes Analyzed
Sr_xLa_{1-x}-TaS_2 (10 at% Sr)	38	134	18
Sr_xLa_{1-x}-TaS_2 (20 at% Sr)	47	133	9
Sr_xLa_{1-x}-TaS_2 (40 at% Sr)	78	216	14
Sr_xLa_{1-x}-TaS_2 (60 at% Sr)	64	195	12

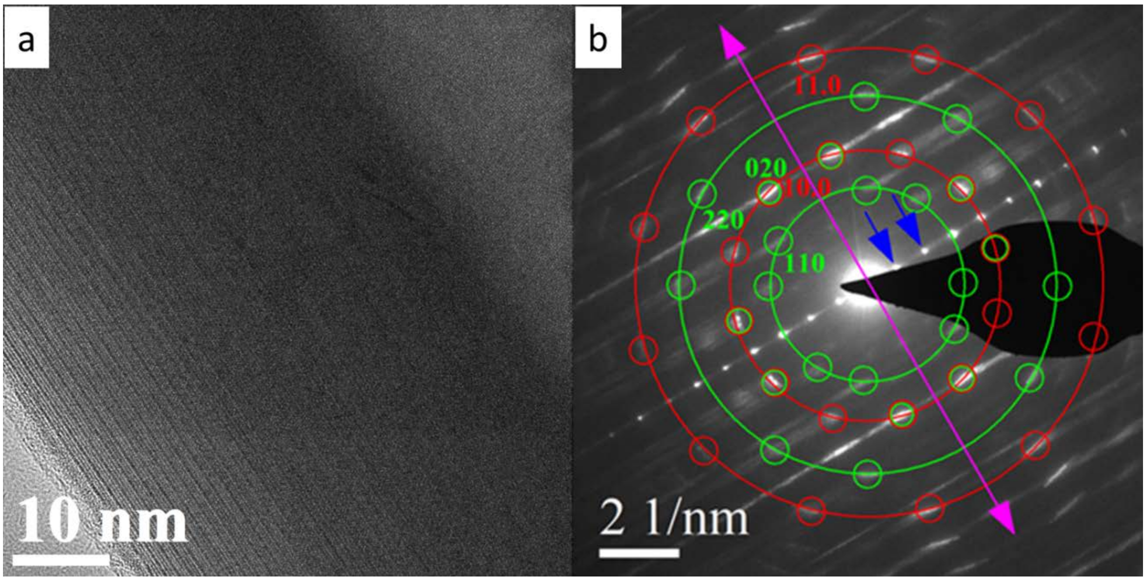

Figure S1. a) HRTEM image of a $Sr_xLa_{1-x}S$-TaS_2 (Sr 60 at%) tube, b) Selected area diffraction showing the orientation relationship between $Sr_xLa_{1-x}S$ layers (green) and TaS_2 layers (red). The tube axis is shown as pink arrow and the basal reflections are marked with blue arrows.

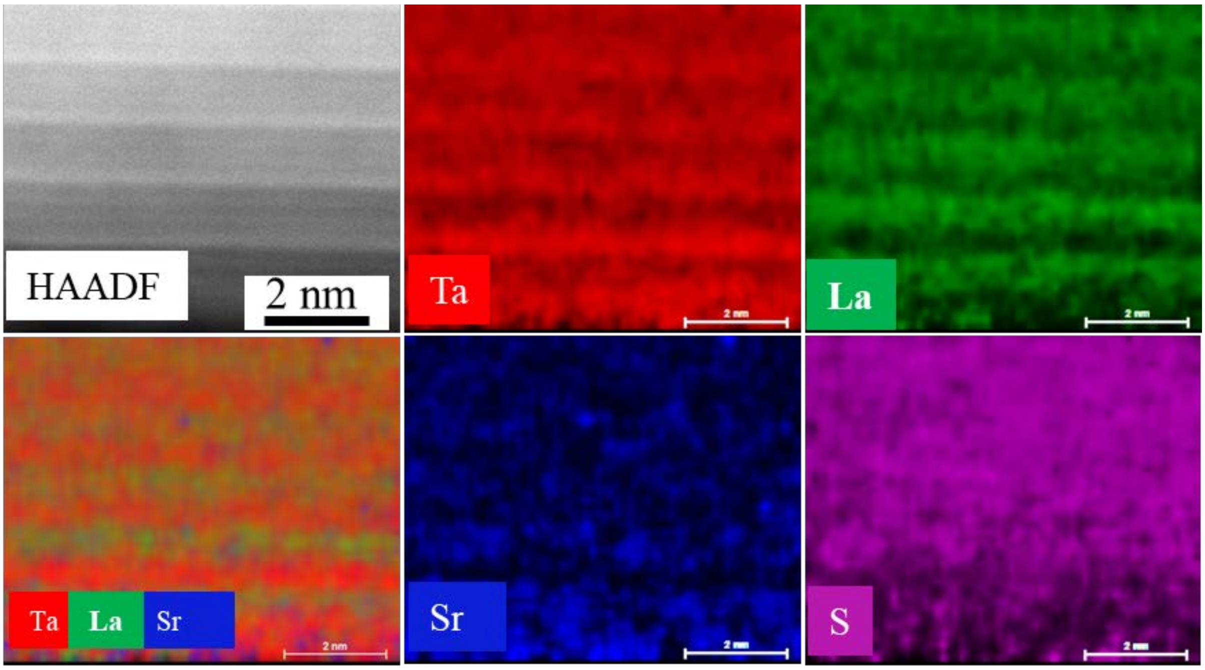

Figure S2. HAADF STEM image and EDX elemental mapping on a $Sr_xLa_{1-x}S-TaS_2$ tube synthesized with Sr 60 at% in the precursor.

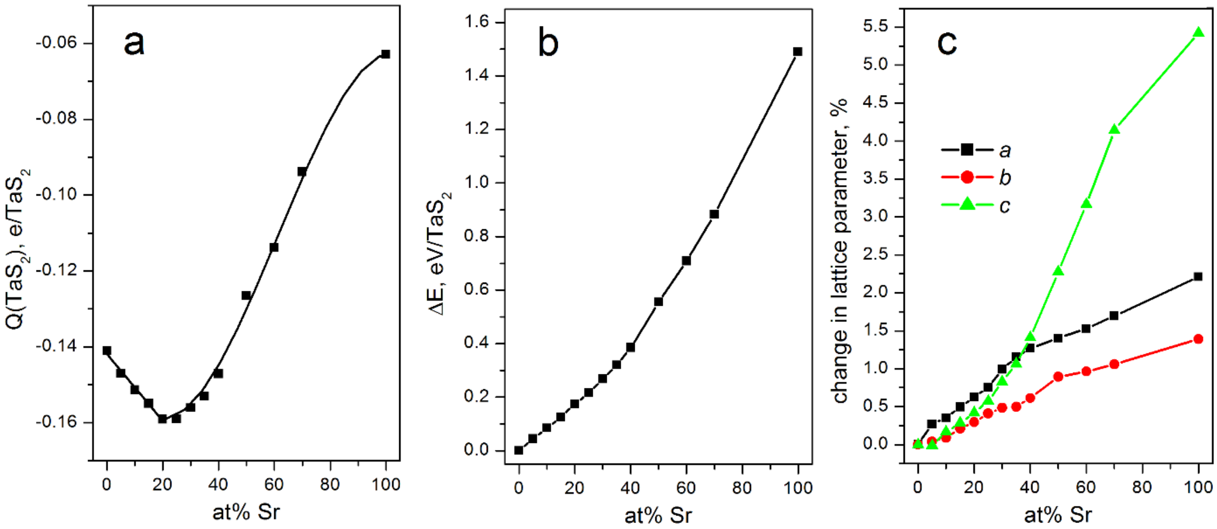

Figure S3. (a) The variations in the amount of charge transfer from $Sr_xLa_{1-x}S$ to TaS_2 within $Sr_xLa_{1-x}S-TaS_2$ misfits (Q), (b) the relative formation energies (ΔE) and (c) the lattice parameters (*a*, *b*, *c*), depending on the Sr content. DFT calculations.

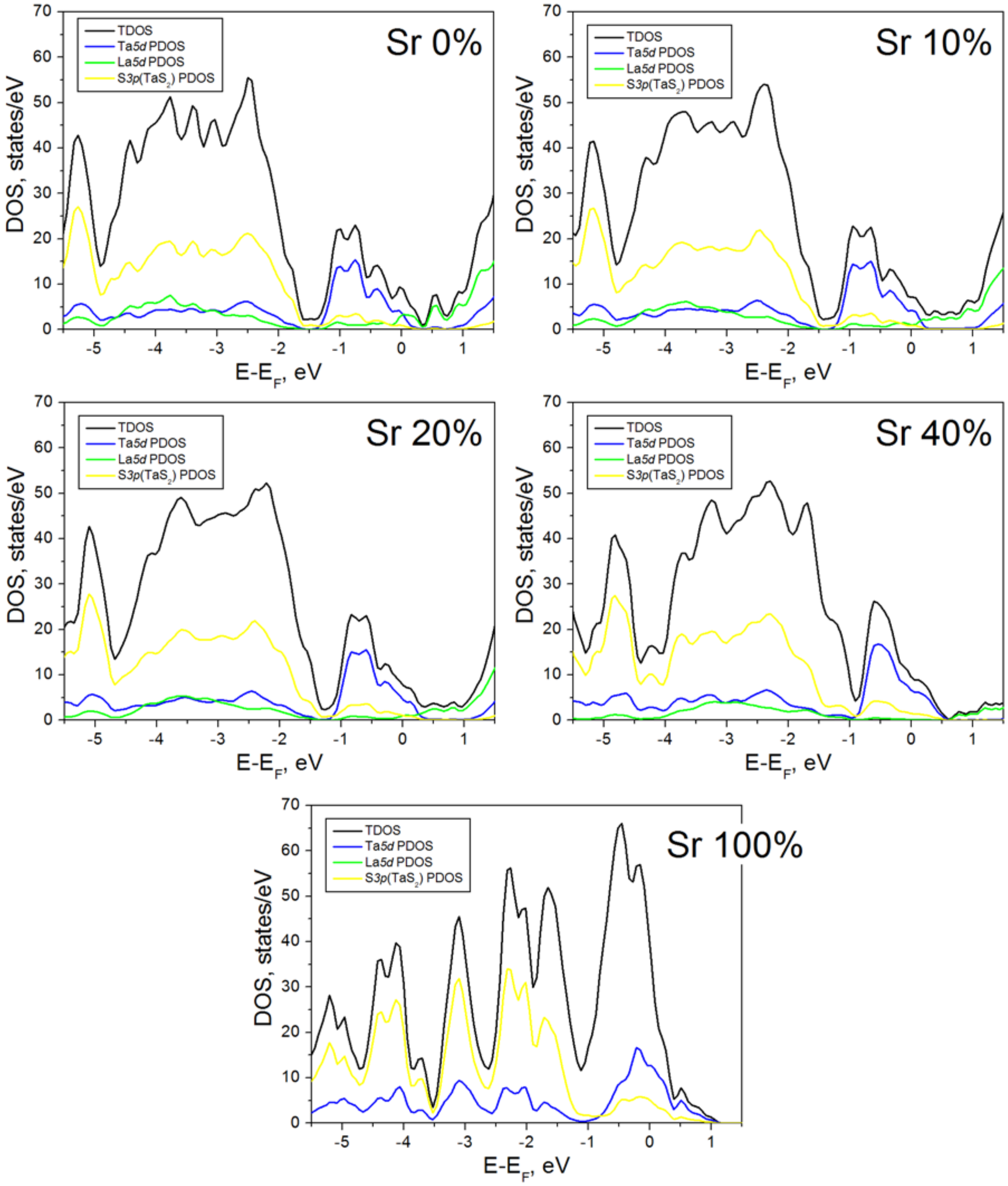

Figure S4. Total and selected partial densities-of-states (DOS) for $Sr_xLa_{1-x}S$-TaS_2 misfits with different Sr content. DFT calculations.
